# Dietary Synbiotic Supplementation Protects Barrier Integrity of Hepatocytes and Liver Sinusoidal Endothelium in a Mouse Model of Chronic-Binge Ethanol Exposure

**DOI:** 10.3390/nu12020373

**Published:** 2020-01-31

**Authors:** Yingchun Han, Bryan Glueck, David Shapiro, Aaron Miller, Sanjoy Roychowdhury, Gail A. M. Cresci

**Affiliations:** 1 Department of Inflammation & Immunity, Lerner Research Institute, Cleveland Clinic, Cleveland, OH 44195, USA; hany2@ccf.org (Y.H.); glueckb@ccf.org (B.G.); shapird2@ccf.org (D.S.); millera25@ccf.org (A.M.); roychos@ccf.org (S.R.); 2 Department of Pediatric Gastroenterology, Cleveland Clinic Children’s Hospital, Cleveland, OH 44195, USA; 3 Center for Human Nutrition, Digestive Disease and Surgery Institute, Cleveland Clinic, Cleveland, OH 44195, USA

**Keywords:** synbiotic, butyrate, liver sinusoidal endothelial cell, ethanol, gut microbiota, liver

## Abstract

Alcohol overconsumption disrupts the gut microbiota and intestinal barrier, which decreases the production of beneficial microbial metabolic byproducts and allows for translocation of pathogenic bacterial-derived byproducts into the portal-hepatic circulation. As ethanol is known to damage liver sinusoidal endothelial cells (LSEC), here we evaluated dietary supplementation with a previously studied synbiotic on gut microbial composition, and hepatocyte and LSEC integrity in mice exposed to ethanol. We tested a chronic-binge ethanol feeding mouse model in which C57BL/6 female mice were fed ethanol (5% vol/vol) for 10 days and provided a single ethanol gavage (5 g/kg body weight) on day 11, 6 h before euthanasia. An ethanol-treatment group also received oral supplementation daily with a synbiotic; and an ethanol-control group received saline. Control mice were pair-fed and isocalorically substituted maltose dextran for ethanol over the entire exposure period; they received a saline gavage daily. Ethanol exposure decreased gut microbial abundance and diversity. This was linked with diminished expression of adherens junction proteins in hepatocytes and dysregulated expression of receptors for advanced glycation end-products; and this coincided with reduced expression of endothelial barrier proteins. Synbiotic supplementation mitigated these effects. These results demonstrate synbiotic supplementation, as a means to modulate ethanol-induced gut dysbiosis, is effective in attenuating injury to hepatocyte and liver endothelial barrier integrity, highlighting a link between the gut microbiome and early stages of acute liver injury in ethanol-exposed mice.

## 1. Introduction

Overconsumption of alcohol can contribute to chronic liver diseases, resulting in hepatic steatosis, alcoholic hepatitis, with progression to fibrosis, cirrhosis, and hepatocellular carcinoma (reviewed elsewhere [1]). Chronic ethanol exposure leads to defenestration in liver sinusoidal endothelial cells (LSEC) [2], which allows for sinusoidal blood and contents to associate with hepatocytes, hepatic stellate cells, and Kupffer cells [3]. Acute toxin exposure, such as acetaminophen and ethanol induces gaps in the LSEC and induces centrilobular sinusoidal collapse which reduces blood flow, and impairs the microcirculatory exchange of nutrients and clearance of waste products [4]. The effect of chronic-binge ethanol exposure on the interaction between adherence junctions of the hepatocytes and the neighboring endothelial cells remains unclear.

Oxidative metabolites of ethanol, such as acetaldehyde and reactive oxygen species (ROS), are main contributors to alcoholic liver injury (ALD). ROS generation has been observed in numerous hepatic cell types during ethanol exposure, but the role of ROS in ALD development is uncertain. Oxidation of alcohol in the liver yields acetaldehyde by mainly alcohol dehydrogenase and to a lesser extent Cytochrome P450 family 2, subfamily E, polypeptide 1 (CYP2E1). Acetaldehyde is then oxidized into acetate by aldehyde dehydrogenase. Acetaldehyde causes direct hepatocyte damage and also forms adducts with proteins and DNA [5]. Acetaldehyde–derived advanced glycation end-products (AA-AGE) are produced from acetaldehyde and have properties similar to adducts formed by non-enzymatic glycation of sugar (AGE adducts), which are linked with non-alcoholic fatty liver disease [6]. AA-AGEs induce oxidative stress and generate ROS in rat hepatic stellate cells [5]. Similarly, patients with ALD demonstrated intense hepatic staining for AA-AGEs, 4-hydroxy-2-nonenal (4-HNE), an oxidative stress marker, and steatosis, which is correlated with disease severity as compared to those without ALD [5]. These data suggest ROS generation by AA-AGEs contribute to the pathophysiology of ALD.

Advanced glycation end-products induce tissue injury by exerting direct physiochemical effects, and indirect biologic effects, mediated by cell surface receptors. Binding of AGE to these receptors and downstream signaling and transcriptional effects have been shown to affect tissue injury in several pathophysiologic conditions, such as vascular disease, diabetes, cancer, and neurodegeneration [7,8]. These receptors are upregulated by their ligands, with the consequent propagation and amplification of effects. The first identified receptor for AGE (RAGE), upon binding with its ligand, is associated with cell activation and proinflammatory signaling via receptor-mediated generation of ROS [7]. Galectin-3, originally identified as Mac-2, has ubiquitous localization and functions within the cell and within the liver [8]. Galectin-3 is involved in the uptake and removal of AGEs, playing an anti-inflammatory role [8,9]. Thus galectin-3 and RAGE appear to exert opposite actions as AGE receptors, participating in the pathogenesis of metabolic disorders in opposite ways.

In addition to endogenous production, AGEs may also be ingested, which can contribute to their accumulation. AGEs are found in cigarette smoke, and may be formed in foods depending upon heat treatment. Since only 10%-30% of ingested AGEs are absorbed, the unabsorbed AGEs may interact with the gut microbiota. Human investigations show ingestion of AGEs decrease beneficial gut microbiota abundance, but not alpha-diversity [10]. Animal studies show contraction of beneficial bacteria (e.g., Bacteroidetes, *Lactobacilli, Bifidobacteria*) with high-AGE dietary exposure, but other studies are contradicting [10]. Ethanol ingestion induces gut dysbiosis [11,12] and depletes short-chain fatty acid levels [13,14], but whether this is associated with liver vascular injury and AGE/AA-AGE is unknown.

We previously found supplementation with a designer synbiotic protects against gut–liver injury induced by chronic-binge ethanol exposure in mice [14]. The synbiotic consisted of a butyrate-producing and anti-inflammatory commensal microbe, *Faecalibacterium prausnitzii*, and a butyrate-yielding prebiotic (potato starch). Butyrate, a fermentation byproduct of commensal gut microbiota, supports the gut barrier and immune function, and is anti-inflammatory [15]. We found that synbiotic supplementation protects the expression of intestinal epithelial barrier proteins in the proximal colon and decreases hepatic markers for inflammation (tumor necrosis factor-alpha, TNF-α) and oxidative stress (4-HNE), and decreases steatosis induced by chronic-binge ethanol exposure [14]. These findings were associated with protection in the protein expression of butyrate transporters in the proximal colon and liver. Our prior work also shows oral butyrate (tributyrin) supplementation protects the expression of endothelial and immune cell markers in the proximal colon of mice exposed to chronic-binge ethanol [16]. Therefore, here we tested the hypothesis that supplementation with this synbiotic would be protective against chronic-binge ethanol-induced hepatocyte and LSEC injury in mice.

## 2. Materials and Methods

Female C57BL/6 mice (10–12 weeks old) were purchased from Jackson Labs (Bar Harbor, ME, USA). Lieber-DeCarli ethanol and control diets were purchased from Dyets (Bethlehem, PA, USA). *Faecalibacterium prausnitzii* 27766 was purchased from ATCC (Manassas, VA, USA); potato starch (S2004; CAS Number 9005-25-8), sodium butyrate, and lipopolysaccharide (LPS) were purchased from Sigma-Aldrich (St. Louis, MO, USA); human umbilical vein endothelial cells (HUVEC) were purchased from Lonza (Walkersville, MD, USA). Antibodies were from the following sources: Antiplatelet endothelial cell adhesion molecule (PECAM-1/CD31), von Willibrand Factor (vWF), Type IV Collagen, Beta-catenin, Claudin-5, and vascular endothelial cadherin (VE-cadherin) were from Abcam (Cambridge, MA, USA); Type I Collagen was from SouthernBiotech (Birmingham, AL, USA); epithelial cadherin (E-cadherin) was from Thermofisher (Rockford, IL, USA); galectin-3 was from Cedarlane (Burlington, NC); receptor of advanced glycation end-products (RAGE) was from Novus Biologicals (Centennial, CO, USA); F4/80 was from Bio-Rad (Hercules, CA, USA); HSC70 was from Santa Cruz Biotech (Dallas, TX, USA); Alexa Fluor 488 and 568 from Invitrogen (Carlsbad, CA, USA). All primers for quantitative real-time reverse transcription polymerase chain reaction (qRT-PCR) were synthesized by Integrated DNA Technologies (Coralville, IA, USA).

### 2.1. Ethanol Exposure Model and Dietary Supplementations

The Cleveland Clinic Institutional Animal Care and Use Committee approved all animal procedures. Housed in cages (2 animals/cage) with microisolator lids, mice were randomized into ethanol-fed and pair-fed groups and then adapted to a control liquid diet for five days. The ethanol-fed group was allowed free access to a diet containing 5% (*v*/*v*) ethanol; control mice were pair-fed diets containing isocalorically substituted maltose dextrins for ethanol over the entire exposure period. The next day mice received an ethanol bolus (5 g/kg body weight) orally and were euthanized 6 h later. Throughout ethanol feeding, a group of mice (ET-Syn) were provided the synbiotic (FP: 6 log10 CFU/10 µL daily + potato starch (C6H10O5) n (PS: 20% *w*/*v*, 20 µL daily) by oral gavage; control mice were gavaged with 0.9% normal saline (PF-S). The composition of the synbiotic was as per prior studies demonstrating beneficial effects [17]. Livers were blanched with saline via the portal vein and then excised. Portions of each liver were either fixed in formalin or frozen in optimal cutting temperature (OCT) compound (Sakura Finetek U.S.A., Inc., Torrance, CA, USA) for histology, frozen in RNAlater (Qiagen, Valencia, CA, USA), or flash frozen in liquid nitrogen and stored at −80 °C until further analysis.

### 2.2. Fecal Microbiota Analysis

Cecal contents were stored at −20 °C prior to DNA extraction. Subsequently, 0.20–0.25 g of stool was used for DNA extraction with the QIAamp PowerFecal DNA Kit (QIAGEN GmbH, Hilden, Germany). Negative controls consisting of sterile water were used with each round of extractions to ensure no contamination in the extraction reagents was present. All extractions were verified through gel electrophoresis and a Nanodrop Spectrophotometer (Thermo Scientific) and only samples with a positive band and DNA concentration >20ng/μL were used for sequencing. No negative controls and all stool samples exhibited the presence of DNA by these criteria.

Extracted DNA was sent for sequencing of the hypervariable V4 region of the 16S rRNA gene at Argonne National Laboratory (Chicago, IL) on an Illumina MiSeq, using the primers 515F [18] and barcoded samples were multi-plexed on a single lane for 150 base pair, pair-ended sequencing. To analyze sequencing data, raw reads were demultiplexed and quality-controlled using the default parameters in QIIME [19]. An open reference strategy was used to classify operational taxonomic units (OTUs) with 97% sequence homology compared to the Greengenes dataset [20]. Following taxonomic assignment, sequences were removed if they were identified as chimeras, chloroplasts, mitochondria, or had <10 representations across the whole dataset. Prior to subsequent comparative analyses, data were normalized with a negative binomial Wald test through the DESeq2 package in R [21].

The phylogenetic diversity was quantified for each group, with group means compared with a two-way ANOVA, followed by a post-hoc, Holm’s-corrected, paired t-test. For β-diversity, unweighted UniFrac distances were calculated and statistical analyses between groups were conducted with a two-way Permanova analysis with 999 permutations [22]. Differential abundance analysis was calculated as a two-way Wald test (log2 fold change/standard error), with a false discovery rate correction for multiple comparisons in the DESeq2 statistical package [21].

### 2.3. Human Umbilical Vein Endothelial Cell Culture (HUVEC)

The HUVEC cell line was maintained in Endothelial Cell Growth Medium (Lonza, Walkersville, MD, USA). Cells were grown in fibronectin coated 75-cm^2^ T-flasks (Fisher, St. Louis, MO, USA) at 37 °C/5% CO_2_. The medium was changed three times per week, every 7 to 10 days cells were split, and experiments were conducted with cells that were within four passages of each other. Confluent monolayers were harvested on ice with a cell lifter following a wash with ice cold PBS for RNA extraction. HUVEC were seeded at 3 × 10^4^ density into the apical chamber of a two-chamber cell culture system (Costar Corp., Cambridge, MA, USA) containing a 0.4-cm^2^ pore size polycarbonate membrane or into a 24-well plate containing a sterile coverslip. Three days after seeding, the monolayer was exposed to ethanol (40mM), LPS (1 ug/mL), or both, with and without sodium butyrate (2 mM) for 6 hrs. This dosing and timing were based on prior in vivo and in vitro studies [23,24,25]. Ethanol treated monolayers were seeded on culture plates separate from those not exposed to ethanol. All plates (24-well) were sealed with parafilm wrap, during the 6 h incubation period. This was a precaution to minimized ethanol evaporation. HUVEC monolayers seeded on coverslips were then rinsed with PBS and fixed with 4% PFA followed by immunostaining for endothelial barrier proteins.

### 2.4. Measurement of Transendothelial Electrical Resistance (TER)

Determination of endothelial barrier function of HUVEC monolayer and the integrity of barrier proteins formed was assessed by measurements of TER using an EVOM2 Epithelial Vol/Ohm Meter (World Precision Instruments, Sarasota, FL, USA), as described previously [26]. Briefly, following rinsing cells with room temperature PBS, an average of three measurements per transwell were obtained and averaged. Measurements were acquired at 0 h (baseline) and after 6 h of treatment exposures. Experiments were performed in triplicate and repeated five times.

### 2.5. Immunofluescent Staining

The assay was performed according to the protocol previously described [14,16] Liver sections and HUVEC monolayers underwent immunostaining for proteins expressed by vascular and endothelial cells (vWF, CD31, Type IV Collagen, E-Cadherin, VE-Cadherin, Claudin-5, and β-catenin), macrophages (F4/80), and for the receptor for advanced glycation end-products (RAGE) in the liver. Specific immunofluorescent positive staining was compared to sections incubated with phosphate-buffered saline (PBS) as a negative control; in these studies no positive staining was seen with PBS incubation. A single investigator blinded to treatments viewed the stained sections. All images presented represent at least three images per tissue section and 8 to 12 mice per experimental condition. Semi-quantification of positive staining was performed using ImagePro plus software (Media Cybernatics, Silver Spring, MD, USA). Images were taken Keyence B2-X810 All-in-one Fluorescence Microscope (Itasca, IL, USA) at 10× or 20×.

### 2.6. Western Blot

Liver homogenates were prepared, and protein concentrations were determined for immunoblotting, as previously described [14]. Protein (30 µg/20 µL) was resolved on 6%–10% polyacrylamide gels and transferred to polyvinylidene fluoride membranes. Antibodies specific for Galectin-3, E-cadherin, β-catenin, CD31, and Type I and IV Collagen were probed; and HSC70 served as the loading control. At least 6 to 8 mice per treatment group were analyzed. Immuno-reactive protein expression was detected using enhanced chemiluminescence and signal intensities were determined by densitometry using ImageJ software (NIH, Bethesda, MD, USA), as previously described [14].

### 2.7. Quantitative Real-time Reverse Transcription Polymerase Chain Reaction

Total RNA was isolated from liver from at least 8 to 12 mice per experimental condition, and 2 µg of total RNA was reverse transcribed, as previously described [17]. A QuantStudio 5 analyzer (Applied Biosystems, Foster City, CA, USA) was used to perform real-time PCR amplification with PowerSyBR qRT-PCR kits (Applied Biosystems) for primers: Matrix Metallopeptidase 9: FWD: GCG CCA CAG CCA ACT ATG; REV: TGG ATG CCG TCT ATG TCG TCT TTA; TIMP1: FWD: GCA ACT CGG ACC TGG TCA TAA; REV: CGG CCC GTG ATG AGA AAC T

The comparative threshold (Ct) method was used to determine the relative amount of target mRNA to the values of the housekeeping gene, glyceraldehyde 3-phosphate dehydrogenase (GAPDH). Graphs are represented as fold change relative to saline treated pair-fed mice (the control group).

### 2.8. Statistical Analysis

All data are expressed as the mean ± standard error of the mean (SEM) with *n* = 8–16 mice per treatment groups. A Student t-test was used for the parametric analysis of two groups; analysis of variance was used for a comparison of multiple groups with a Tukey’s post hoc test for multiple comparisons. Data were log-transformed to obtain a normal distribution as needed. Statistical significance was defined as *p* < 0.05. The analysis was performed using Prism software Version 5.02 (GraphPad Software, San Diego, CA, USA).

## 3. Results

### 3.1. Synbiotic Effects on Gut Microbiota During Chronic-Binge Ethanol Exposure

**Figure 1:**
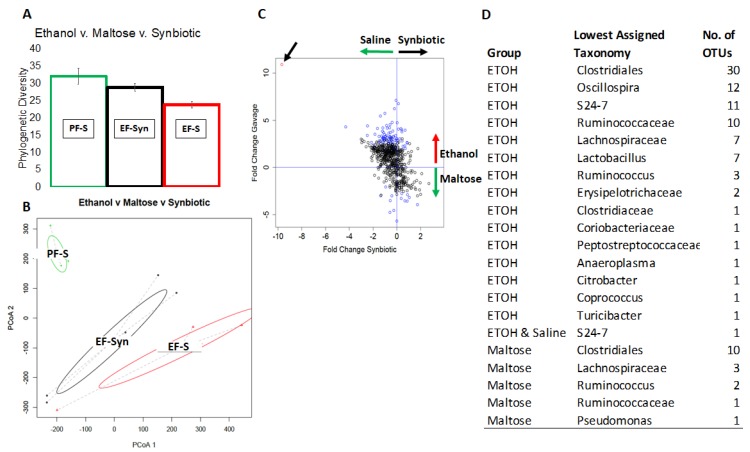
The effect of ethanol and synbiotic on the microbiota. Mice were fed a liquid diet containing ethanol (5% *v*/*v*) or pair-fed a diet with maltose dextrin isocalorically substituted for ethanol for 10 days. Mice were randomized into groups and supplemented with a butyrate-targeting synbiotic or saline. On day 11, mice were treated with a single 5 g/kg gavage of ethanol. At 6 h post-gavage, the cecum was dissected and flash frozen and kept at −20 °C until analysis (see methods). (**A**) Ethanol exposure decreased the overall phylogenetic diversity of the cecal microbiota (*p* = 0.001), which was partially recovered by synbiotic supplementation; (**B**) ethanol significantly altered microbiota community composition and structure, as assessed by a weighted UniFrac analysis followed by PERMANOVA (*p* = 0.007). The ethanol-synbiotic group clusters away from animals only receiving ethanol indicated some recovery, but the difference was not significant; (**C)** differential abundance analysis, executed as a negative binomial Wald test, revealed operational taxonomic units (OTUs) significantly enriched in either the maltose or ethanol groups (blue circles) or both ethanol and saline (red circle); (**D**) list of microbial taxa enriched in either the maltose, ethanol, or saline groups. *n* = 4-6 mice per treatment group.

A total of total of 951,266 high quality sequences were generated from 20 samples, which amounted to 47,561 +/− 2734 reads/sample and 1310 unique OTUs across the whole dataset. All samples were dominated by the *Firmicutes* phylum. Statistical analysis revealed that phylogenetic diversity was lower in mice fed ethanol compared to those fed maltose (*p* = 0.001–0.008) (Figure 1A). The effect of ethanol was partially attenuated by the synbiotic, however the effect was not significant. Additionally, ethanol had a significant impact on overall community composition, as determined by PERMANOVA analysis of the β-diversity (*p* = 0.007–0.036) (Figure 1B). The effect of ethanol on community composition was partially, but not significantly, attenuated by the synbiotic. Two-way differential abundance analysis revealed that the *Clostridiales, Ruminococcaceae*, and *Oscillospira* were most differentially abundant in mice given a either ethanol or maltose (Figure 1C,D).

### 3.2. Synbiotic Maintained Sinusoidal Macrophage Population and Adherens Junction Protein Expression

**Figure 2:**
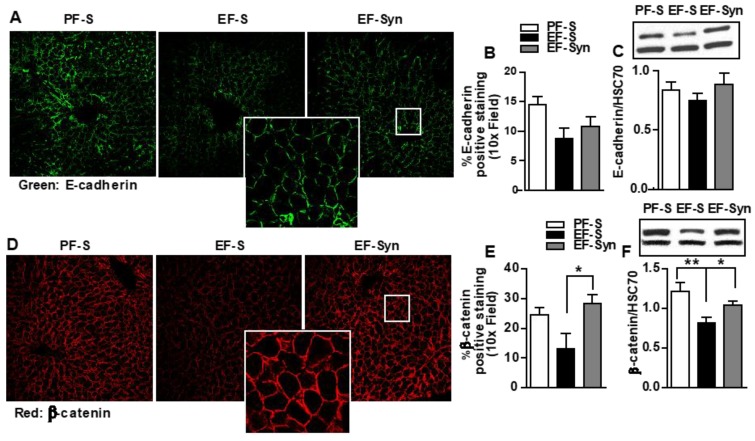
Cellular junctional proteins in mouse liver. Mice were treated as described in Figure 1. Liver sections that were flash frozen for immunoblotting or fixed in formalin and paraffin embedded for immunohistochemical analysis were probed for the following: (**A**–**C**) E-cadherin (green); and (**D**–**F**) β-catenin (red). All images were acquired using a 10x objective, and a selected area was cropped and enlarged. Images are representative of at least replicate images captured per mouse in 8 to 12 mice per treatment group. Immunofluorescent images were semi-quantified, and immunoblot band densities were analyzed using ImageJ software and normalized to HSC70. Values represent means ± SEM. * *p* < 0.05; ** *p* < 0.001. Pair-fed saline (PF-S); Ethanol-fed saline (EF-S); Ethanol-fed synbiotic (EF-Syn).

As a major constituent of adherens junctions, E-cadherin forms cell–cell interactions between epithelial cells and is expressed by hepatocyte and biliary epithelial cells. The specific loss in E-cadherin in liver epithelial cells is associated with periportal fibrosis, periportal inflammation, and liver cancer progression [27]. Intracellulary, E-Cadherin binds to catenins, which links it to the actin cytoskeleton. In hepatocytes, β-catenin is expressed at the cell surface throughout the hepatic lobule, and serves as an intracellular signal transducer in the WNT signaling pathway, in which alterations in its activity are associated with liver diseases [28]. LSEC are exposed to highly oxygenated arterial blood mixed with the portal blood derived from the gut on their sinusoidal side, and interact with hepatic stellate cells and hepatocytes on their abluminal side [3], therefore ethanol-induced gut dysbiosis and intestinal barrier compromise could expose hepatocytes to toxins, such as bacterial byproducts (e.g., LPS) coming from the gut. Disruption in the hepatocyte epithelial barrier could interrupt their function [29]. Therefore, we tested for the expression of hepatocyte epithelial barrier protein by immunostaining and immunoblotting. Hepatic intensity staining for both E-cadherin (Figure 2A,B) and β-catenin (Figure 2D,E) was localized to the epithelial barrier in pair-fed mice. Ethanol exposure decreased both the intensity of the staining and its continuity on the epithelial barrier for β-catenin. This was mitigated in mice that received synbiotic supplementation (Figure 2D,E). Immunoblotting also showed decreased β-catenin in the ethanol-saline group compared to those pair-fed or supplemented with the synbiotic (Figure 2F). Ethanol did not affect the expression of E-cadherin as demonstrated by immunohistochemistry and immunoblotting (Figure 2A–C).

### 3.3. Synbiotic Modulated Immune Cell Compartmentalization in the Liver

**Figure 3:**
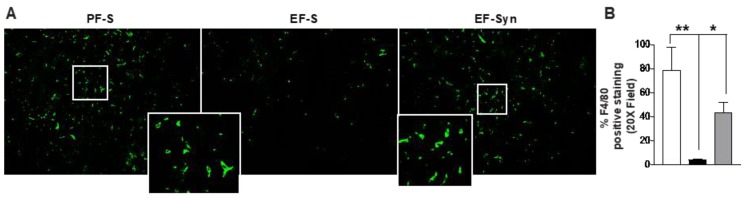
Macrophage staining in mouse liver. Mice were treated as described in Figure 1. Paraffin-embedded liver sections were immuno-stained for the (**A**,**B**) pan-macrophage marker F4/80. Images were acquired using 20× objective, and a selected area was cropped and enlarged. Images are representative of at least replicate images captured per mouse in 8 to 12 mice per treatment group. Immunofluorescent images were semi-quantified using Image Pro Plus software. Values represent means ± SEM. * *p* < 0.05; ** *p* < 0.001. Pair-fed saline (PF-S); Ethanol-fed saline (EF-S); Ethanol-fed synbiotic (EF-Syn).

Reduced expression of adherens junctional proteins in hepatocytes suggested the impairment of sinusoidal fenestrae following chronic-binge ethanol exposure. Since hepatic macrophages reside in the hepatic sinusoid, we hypothesized that ethanol-induced disruption of hepatocyte adherens junctional proteins would be associated with a reduction in numbers of F4/80+ hepatic macrophages. Indeed, ethanol feeding reduced the expression of F4/80 which was protected with synbiotic supplementation (Figure 3A,B), similar to previous reports by Bertola et al. [30].

### 3.4. Decreased AGE Receptor Expression with Synbiotic Supplementation

**Figure 4:**
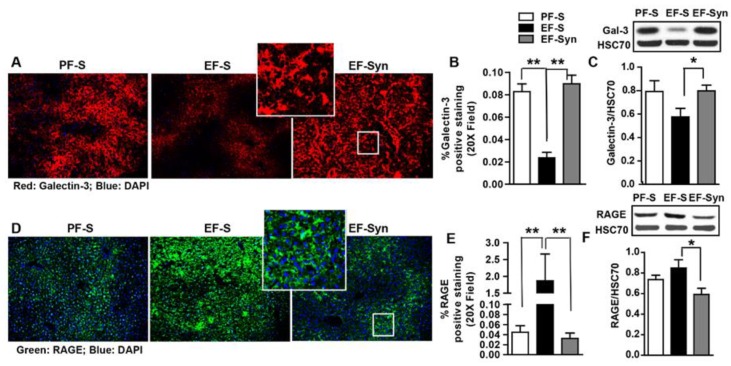
Hepatic expression of advanced glycation end-product (AGE) receptors. Mice were treated as described in Figure 1. Liver was collected and flash frozen for immunoblotting or fixed in formalin and paraffin embedded for immunohistochemical analysis. The liver sections were probed for the following: (**A**–**C**) Galectin-3 (red: Gal-3); (**D**–**F**) the receptor for advanced glycation end-products (green: RAGE). All images were acquired using a 20x objective, and a selected area was cropped and enlarged. Images are representative of at least replicate images captured per mouse in 8 to 12 mice per treatment group. Immunofluorescent images were semi-quantified, and immunoblot band densities were analyzed using ImageJ software and normalized to HSC70. Values represent means ± SEM. * *p* < 0.05; ** *p* < 0.001. Pair-fed saline (PF-S); Ethanol-fed saline (EF-S); Ethanol-fed synbiotic (EF-Syn).

Advanced glycation end-products (AGE) accumulate in metabolic disorders, such as hepatic steatosis, which is the initiating condition in ALD. Plasma AGEs increase markedly in patients with end-stage liver disease, indicating the important role of the liver in catabolizing AGEs [31]. Uptake of AGE in liver endothelial, Kupffer, and parenchymal cells accounted for roughly 60%, 25%, and 10%-15%, respectively, of hepatic elimination [31]. Since we previously reported that synbiotic supplementation protected against hepatic steatosis and markers of oxidative stress [14], we analyzed liver sections in mice for the protein expression of receptors for AGE (RAGE) and galectin-3 by immunostaining and immunoblotting. Decreased hepatic expression of galectin-3 was observed in mice exposed to ethanol-saline. Comparatively, pair-fed mice and ethanol/synbiotic supplemented mice exhibited increased galectin-3 expression, which localized in LSEC and Kupffer cells, as previously described [8] (Figure 4A,B). The pattern of galectin-3 protein expression was confirmed with immunoblotting (Figure 4C). In an opposing expression pattern, we found a clear increase in staining and immunoblotting for RAGE in mouse livers exposed to ethanol-saline (Figure 4D,F), with a comparatively lower RAGE expression for pair-fed and synbiotic supplemented mice. As previously reported, RAGE expression was localized to hepatocytes surrounding the central vein (Figure 4D). These data suggest mice only exposed to ethanol increased AGE/AA-AGE presence with decreased capacity to for their clearance due to reduced galectin-3 expression coupled with impaired LSEC.

### 3.5. Synbiotic Protected Hepatic Vasculature and Sinusoidal Framework during Ethanol Exposure.

**Figure 5:**
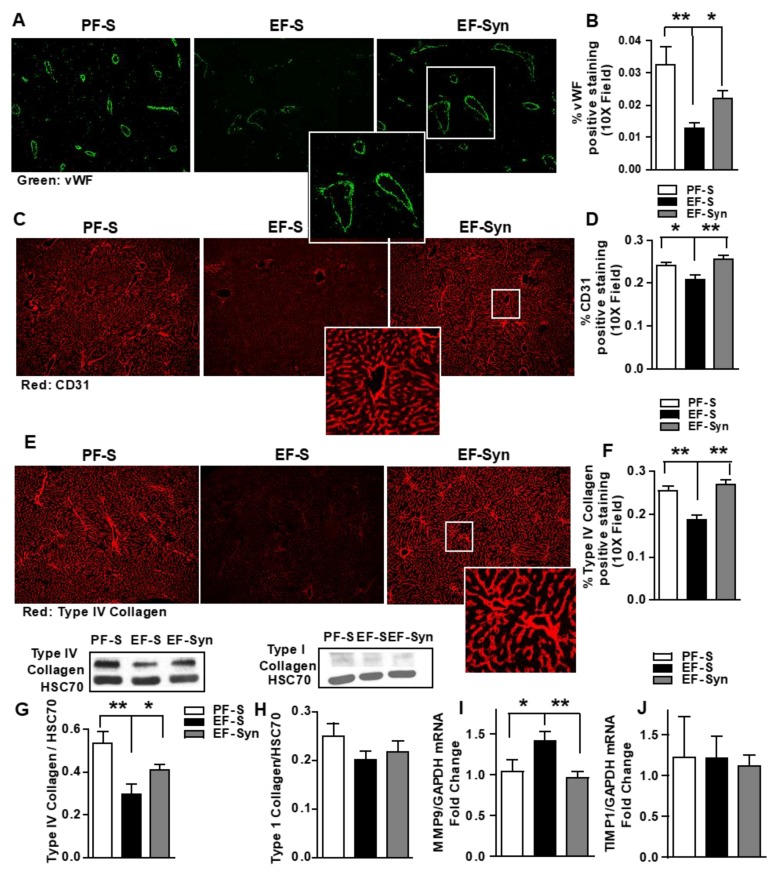
Effect of synbiotic on hepatic vasculature. Mice were treated as described in Figure 1. Upon euthanasia, the liver was dissected and flash frozen for immunoblotting or fixed in formalin and paraffin embedded for immunohistochemical analysis, histology, or RNA was prepared and used for qRT-PCR analysis. The liver sections were probed for the following: (**A**,**B**) von Willebrand Factor (vWF; green); (**C**,**D**) CD31 (red); (**E**–**G**) Type IV Collagen (red); (**H**) Type I Collagen; (**I**) MMP9 mRNA and (**J**) TIMP1 mRNA expression in liver, and presented as fold change. All images were acquired using a 10× objective, and a selected area was cropped and enlarged. Images are representative of at least replicate images captured per mouse in 8 to 12 mice per treatment group. Immunofluorescent images were semi-quantified, and immunoblot band densities were analyzed using ImageJ software and normalized to HSC70. Values represent means ± SEM. * *p* < 0.05; ** *p* < 0.001. Pair-fed saline (PF-S); Ethanol-fed saline (EF-S); Ethanol-fed synbiotic (EF-Syn).

Most complications leading to morbidity in metabolic syndrome are of vascular origin, therefore it is of interest that non-alcoholic fatty liver disease is associated with hepatic microvascular dysfunction and increased AGE [31]. Since Kupffer cells and LSEC are major cellular sites of AGE uptake and clearance, AGE/AA-AGE accumulation in the liver could be associated with hepatic microvascular damage and ALD. Thus, we assessed for the expression of vWF, CD31, Type IV, and Type I collagen by immunohistochemistry and Western blotting. Hepatic intensity staining for vWF (Figure 5A,B), CD31 (Figure 5C,D), and Type IV Collagen (Figure 5E,F) was reduced in mice exposed to chronic-binge ethanol compared to the pair-fed control mice. Reduced staining intensity induced by ethanol exposure was mitigated with synbiotic supplementation. CD31 staining pattern was similar to that of Type IV collagen. Immunoblotting for Type IV collagen protein expression was also reduced in the ethanol-saline mice compared to those pair-fed and supplemented with the synbiotic (Figure 5G). Immunoblotting for Type I Collagen protein expression showed minimal expression in all treatment groups, with no differences between groups (Figure 5H).

Matrix metalloproteinases (MMP) are the main enzymes implicated in extracellular matrix degradation, and tissue inhibitors of metalloproteinases (TIMP) control the proteolytic properties of MMPs. (reviewed elsewhere [32]) Factors which increase MMPs are inflammatory cytokines, hormones, and growth factors. Alterations in MMP–TIMP balances are linked with pathologies. MMP-9 is detected in the vascular areas of livers, but virtually absent in naïve livers [33]. As Type IV collagen is a targeted substrate for MMP9, [32,33] we analyzed the hepatic expression of MMP9 mRNA and TIMP1 as a potential link to the differential expression Type IV collagen expression amongst the mouse groups. Here, we show that MMP9 mRNA increased in mice exposed to chronic-binge ethanol compared to those pair-fed and supplemented with synbiotic (Figure 5I), and that TIMP1 mRNA expression was similar between all mouse groups (Figure 5J). These data suggest that Type IV collagen staining is supportive of the hepatic vasculature and sinusoidal framework rather than an early indicator of fibrosis.

### 3.6. Butyrate Attenuated Ethanol- and LPS-Induced Redistribution of Tight Junction Proteins and Decreased Transendothelial Resistance (TER) in HUVEC Monolayers

The synbiotic consisted of a butyrate-producing commensal bacteria and a butyrate-yielding prebiotic. Since our prior work found protection of butyrate transporter expression in the liver of mice exposed to chronic-binge ethanol and synbiotic supplementation [14], here we tested the direct effect of butyrate on endothelial barrier integrity. To test butyrate’s (2 mM, 6 h) direct effect on endothelial barrier integrity during ethanol (40 mM, 6 h) and/or LPS (1 ug/mL, 6 h) exposure, we utilized fully differentiated HUVEC monolayers and evaluated alterations in barrier protein expression and TER. We tested this butyrate dosing and timing based on prior reports [24].

**Figure 6:**
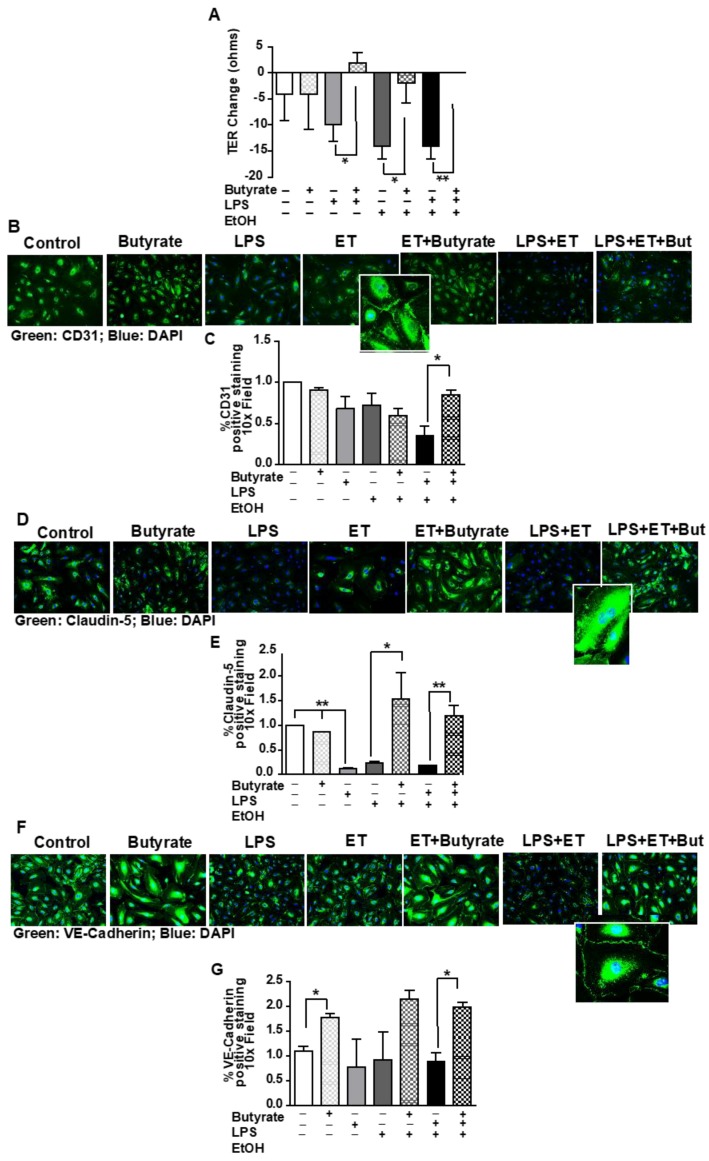
Effect of synbiotic on human umbilical vein endothelial cell culture (HUVEC) integrity to ethanol and/or LPS exposure. Confluent HUVEC monolayers seeded on transwells and/or on glass coverslips in a 24-well culture plate were exposed to ± ethanol (40 mM), ± LPS (1 µg/mL), and ± sodium butyrate (2 mM) on the apical surface for 6 h. (**A**) Transendothelial resistance (TER-ohms) was measured at baseline and 6 h after treatment using a World Precision volt/ohm meter electrical resistance system. TER values represent changes between baseline and 6 h exposures. Data are the mean ± SEM of five independent experiments performed in duplicate. Immunostaining was performed for (**B**,**C**) CD31 (green); (**D**,**E**) Claudin-5 (green); and (**F**,**G**) VE-cadherin (green). All images were acquired using a 10x objective, and a selected area was cropped an enlarged. Images are representative of at least replicate images captured in three independent experiments. Immunofluorescent images were semi-quantified. ** *p* < 0.001; * *p* < 0.05. Lipopolysaccharide (LPS); Ethanol (ET); Butyrate (But).

When cultured with ethanol and/or LPS, barrier protein (CD31, Claudin-5, VE-Cadherin) staining was diminished with varying effects based on the protein. Claudin-5 expression is unique to endothelial cells [34] and was impacted most by these challenges, with decreased expression with LPS, ethanol, and LPS/ethanol exposure; and butyrate co-treatment protected against these effects (Figure 6D). CD31 and VE-cadherin expression [35], also unique to endothelial cells, decreased with ethanol/LPS exposure, but butyrate treatment protected this expression (Figure 6B,F). Endothelial barrier protein expression was coupled with TER, as ethanol and/or LPS exposure diminished TER (Figure 6A) over the 6 h exposure period. Co-treating the monolayer with butyrate attenuated TER reduction in treated monolayers.

## 4. Discussion

The present study found that mice exposed to chronic-binge ethanol exhibited decreased gut microbial community composition and diversity, as well as disruption in hepatocyte epithelial barrier protein distribution, which was associated with dysregulated responses for receptors for AGEs (RAGE, Galectin-3). Further liver analysis showed that mice only exposed to ethanol presented rarefaction of hepatic vasculature and sinusoidal endothelium. Interestingly, synbiotic supplementation mitigated these effects and also partially restored altered gut microbial phylogenetic diversity and community composition that was diminished by ethanol exposure. Thus, these data link alterations in gut microbiota with initial stages of liver injury initiated by chronic-binge ethanol in mice.

A close functional and bidirectional communication exists between the intestine and the liver, highlighting the important interaction between the host and the gut microbiota. Ethanol, both chronic and acute ingestion, exhibit negative actions on gut microbial diversity and function, decreasing beneficial microbes and their production of beneficial metabolic byproducts, and increasing expansion of opportunistic bacteria and their metabolic byproducts (e.g., LPS) [12,13,36,37,38,39]. We previously reported that chronic-binge ethanol-induced liver injury in mice coincided with depleted luminal butyrate levels, and that synbiotic supplementation protected mice from losses in expression of intestinal epithelial barrier proteins as well as butyrate transporters in the proximal colon and the liver [14]. Butyrate is of high biological importance as it serves as the primary fuel source for colonocytes and modulates immune and inflammatory responses and the intestinal barrier. Through its role as a histone deacytylase inhibitor, butyrate can induce epigenetic changes [15].

The beneficial effect of butyrate in supporting the intestinal epithelial barrier disrupted by ethanol exposure is well-characterized [14,23,40]. While 70% of generated butyrate is metabolized by the colonic epithelium, following transport from the intestinal lumen, butyrate may also enter into circulation via the portal vein. Total short-chain fatty acids (SCFA) concentrations measured in the blood (umol/L) were 375 ± 70 (portal), 148 ± 42 (hepatic), and 79 ± 22 (peripheral vein) [41]. Here we show for the first time that supplementation with a synbiotic, comprised of a butyrate-producing and anti-inflammatory commensal bacteria and a butyrate-yielding prebiotic, preserved both hepatocyte epithelial barrier and supported endothelium barrier integrity. Interestingly, our prior work demonstrated that butyrate transporter expression in the liver was localized predominantly around the portal vein in hepatocytes [14]. The portal vein is the direct entry site into the liver where ethanol and gut microbial bacterial byproducts, such as beneficial SCFA (e.g., butyrate) and deleterious LPS, would enter the liver and interact with LSEC on their sinusoidal side. Thus, ethanol-induced gut dysbiosis and subsequent depleted butyrate generation and SCFA transporter expression could compromise the hosts’ ability to counteract the harmful effects of LPS on LSEC and hepatocytes. Previously, we found that mice supplemented with butyrate (tributyrin) during chronic-binge ethanol exposure exhibited protection of vascular and endothelial barrier protein expression in the proximal colon [16]. Thus, here we confirmed with an in vitro model the direct beneficial effects of butyrate on endothelial barrier integrity disrupted by ethanol and/or LPS. Synbiotic supplementation may also have exhibited these effects by decreasing oxidative stress induced by ethanol. Dietary supplementation with pomegranate [42] and symbiotic [14] suppressed oxidative stress in chronic-binge ethanol feeding in rats and mice, respectively. Oxidative stress has been implicated in the pathogenesis of various cardiovascular diseases [43], and the antioxidant properties of both butyrate and pomegranate may also be contributing to these effects in the liver. Communication between hepatocytes and LSECs is crucial in the healthy liver. LSEC regulate liver inflammation by serving as a barrier separating blood from the rest of the liver. Thus, defenestration of LSEC facilitates plasma substances to interact with cells located in the perisinusoidal space. LPS-induced local and systemic inflammation is associated with liver cirrhosis and end-stage liver disease [38]. Hepatic sinusoids normally lack a basement membrane, although Type IV collagen is a normal matrix component of the space of Disse [39]. Separated from the sinusoids by the space of Disse, hepatocytes can become vulnerable to blood components if the sinusoidal fenestrate are impaired. Hepatocytes are the chief functional cells of the liver, performing metabolic, endocrine, and secretory functions. Here, we demonstrate chronic-binge ethanol exposure compromised hepatocyte epithelial barrier as well as reduced sinusoidal staining for macrophages. Compromised sinusoidal networks may also facilitate activation of hepatic stellate cells within the sinusoidal space, a key initiating event in hepatic fibrogenesis [1]. Here we found that synbiotic supplementation mitigated these effects; and previously, we reported synbiotic supplementation prevented liver injury and hepatic steatosis induced by chronic-binge ethanol exposure in mice [14]. Together, these data suggest ethanol-induced gut dysbiosis is a key driver for early stages of chronic-binge ethanol induced liver injury via LSEC compromise.

Alterations in gut microbes can affect their ability to metabolize xenobiotics (e.g., ethanol), as was demonstrated in germ-free mice that had more efficient xenobiotic metabolism and exhibited higher susceptibility to acute alcohol-induced liver injury relative to conventional mice [44]. Acetaldehyde is the first ethanol metabolic byproduct, which occurs primarily in the liver. Acetaldehyde can cross-react to form hybrid adducts, such as AA-AGEs. Adducts accumulate in perivenous regions in rodents fed alcohol and in the liver of alcoholics [45,46,47]. AGEs have a potential role in the pathogenesis of many chronic diseases with vascular origin, such as atherosclerosis and diabetes [31]. Both ethanol metabolism and AGE generate ROS [1]. Receptors for AGE are upregulated by their ligands. As AGE-proteins are efficiently cleared by scavenger receptor-mediated endocytosis in hepatic sinusoidal cells, disruption in LSEC could lead to AGE accumulation. We found an association with deficient LSEC expression and the expression for AGE receptors; RAGE was induced and galectin-3 was reduced with chronic-binge ethanol exposure, and synbiotic supplementation resulted in the opposite effect. We previously reported that chronic-binge ethanol exposure induced hepatic expression of markers for oxidative stress (4-HNE) and inflammation, which was mitigated with synbiotic supplementation [14]. Taken together, these data suggest synbiotic supplementation during chronic-binge ethanol exposure mitigated compromise in LSEC, which may have supported clearance of AA-AGE and decreased oxidative stress in the liver.

## 5. Conclusions

Here, we demonstrated that dietary supplementation with a targeted synbiotic influenced gut microbial phylogenic diversity and community composition, and that this was associated with protection against chronic-binge ethanol-induced disruption in hepatocyte epithelial and endothelial barrier integrity. These data suggest a targeted nutritional approach to stabilize the gut microbiota may thwart the consequential effects of ethanol in the liver. Further studies interrogating potential mechanisms of specific microbes and metabolic byproducts on endothelial function during ethanol exposure are warranted.
